# Film Thickness and Flow Properties of Resin-Based Cements at Different Temperatures

**Published:** 2013-06

**Authors:** R Bagheri

**Affiliations:** a Dept. of Dental Materials and Biomaterial Research Center, School of Dentistry, Shiraz University of Medical Sciences, Shiraz, Iran.

**Keywords:** Film Thickness, Flow, Resin Cements

## Abstract

**Statement of Problem:** For a luting agent to allow complete seating of prosthetic restorations, it must obtain an appropriate flow rate maintaining a minimum film thickness. The performance of recently introduced luting agents in this regard has not been evaluated.

**Purpose:** To measure and compare the film thickness and flow properties of seven resin-containing luting cements at different temperatures (37**°**C, 25**°**C and10**°**C).

**Material and Methods:** Specimens were prepared from five resin luting cements; seT (SDI), Panavia F (Kuraray), Varioloink II (Ivoclar), Maxcem (Kerr), Nexus2 (Kerr) and two resin-modified glass-ionomer luting cements (RM-GICs); GC Fuji Plus (GC Corporation), and RelyX Luting 2 (3 M/ESPE). The film thickness and flow rate of each cement (n=15) was determined using the test described in ISO at three different temperatures.

**Results:** There was a linear correlation between film thickness and flow rate for most of the materials. Cooling increased fluidity of almost all materials while the effect of temperature on film thickness was material dependent. At 37**°**C, all products revealed a film thickness of less than 25µm except for GC Fuji Plus. At 25**°**C, all cements produced a film thickness of less than 27 µm except for seT. At 10**°**C, apart from seT and Rely X Luting 2, the remaining cements showed a film thickness smaller than 20 µm.

**Conclusion:** Cooling increased fluidity of almost all materials, however. the film thickness did not exceed 35 µm in either condition, in spite of the lowest film thickness being demonstrated at the lowest temperature.

## Introduction

Along with the physico-mechanical properties, other clinically related characteristics such as the film thickness and the flow rate need to be taken into account for the selection of a suitable and durable luting agent. For a luting cement to allow complete seating of the prosthetic restorations, it must obtain an appropriate flow rate maintaining a minimum film thickness. Reduced cement film thickness can also decrease the marginal discrepancies, which in turn reduce the plaque accumulation, periodontal disease and cement dissolution. 

Water based luting agents for the cementation of indirect restoration has long been the cement of choice. When first introduced to the market, the resin luting cements exhibited a greater film thickness than the zinc phosphate cements [1-5]. Van Meerbeek et al. [3] in their study of film thickness and consistency of luting composites,  reported a great diversity among the materials and suggested that the composition of the products are responsible for their poor performance and are in need of optimization. They also speculated that a more adequate method is required for measuring the film thickness of luting composites [3].

However, compared with the zinc phosphate cements, the resin-based cements demonstrated better physical properties, less marginal leakage and greater retention [6]. In their comparative study of the flow rate and hydrolytic degradation of resin cements with zinc phosphate cement, Fraga et al. [1] reported a significantly higher hydrolytic resistance for the resin cements; however no significant difference between their flow rates was observed. 

Recently, the resin cements with new formulations, specially the self-adhesive type, have been developed with better physical and mechanical properties [7-9]. In addition, due to their ability to bond to tooth structures, porcelain and metal alloys, these new resin-based cements have gained more popularity over water- based cements [10]. There has been very few research conducted on the influence of the temperature on the film thickness and flow rate of new self-adhesive resin luting cements. A recent study determined the film thicknesses of the representative resin-modified glass ionomers, resin composites, and self-adhesive resin cements [9]. The results of this study reported that the

resin cements showed a film thickness of 25-μm, which is thinner than the standard film thickness of water-based cements. Therefore, it is not necessary for clinicians to provide additional cement space [9]. 

The purpose of our study was to determine the film thickness and the flow rate of the resin cements, self-adhesive resins and resin-modified glass ionomer luting cements. This study also evaluated the effect of the temperature on these properties. The null hypotheses were: there is no difference among the materials and also that the temperature does not affect the clinical properties of the cements.

## Material and Methods

Seven cements were evaluated which are listed in Table 1. All cements were mixed according to the manufacturers’ instructions, using supplied dispensers and/or auto mixing syringe tips when applicable. Mixing of the encapsulated cements was performed for the recommended time. Each of the seven cements were tested at three different temperatures for both film thickness and fluidity test, so that there were six experimental groups for each cement with five specimens in each group which was achieved statistically. All products were kept in an incubator with a constant temperature accuracy of ±1°C and allowed to adjust to the set temperature for at least one hour before the actual measurement was conducted.

**To measure film thickness**

The film thickness was determined based on ISO 3107 requirements [11]. Two optically flat, square glass plates having a contact surface area of 200mm^2 ^and a thickness of 5 mm were used. The combined thickness of the two optically stacked flat glass plates was measured using a digital micrometer (Absolute Digimatic 500-197, Mitutoyo Corp, Kawasaki, Japan) accurate to 1.25 m. 

**Table 1:** Materials

**Materials**	**Manufacturer**	**Type**	**Matrix**	**Filler Content/Type**	**LOT** **Number(s)**
GC Fuji Plus	GC Corporation, Tokyo, Japan	RM-GIC Acid-base	HEMA	FAS glass	0509221
Rely X Luting2	3M ESPE, St. Paul, MN	RM-GIC Acid-base	HEMA, Bis-GMA	FAS glass, zirconia silica	20060822
Maxcem	Kerr Corporation, Orange, CA	Resin- based self-adhesive, dual cure	GPDM, self-etching**/ **adhering acidic monomer	67 wt % (46 vol %) barium glass, FAS glass, fumed silica	3284928
seT	SDI, Victoria, Australia	Resin-based self-adhesive, dual cure	MPE,UDMA, Photo-initiator	67 wt % (45 vol %)FAS glass, pyrogenic silica	S0905281
Panavia F	Kuraray Medical Inc., Okayama, Japan	Resin-based Conventional Dual cure	10-MDP, HAD	78 wt % silanated (colloidal) silica, silanated barium glass	091180
Nexus2	Kerr Corporation, Orange, CA	Resin-based Conventional	Bis-GMA	70 wt % (47 vol %) FAS glass	3305983
Variolink II	Ivoclar Vivadent AG, Schaan, Liechtenstein	Resin-based Conventional	Bis-GMA, UDMA, TEG-DMA	73.4 wt % (46.7 vol %) barium glass, Ba-Al-fluorosilicate glass, spheroid mixed oxide	J07030 J13724

RM-GIC: Resin-modified glass-ionomer luting cement          GPDM: Glyceroldimethacrylate dihydrogen phosphate

FAS: Fluoroaluminosilicate                                                    HAD: Hydrophobic aromatic dimethacrylate

10-MDP: 10-Methacryloxydecyl dihydrogen phosphate        MPE: Methacrylated phosphoric esters

**Figure 1:**
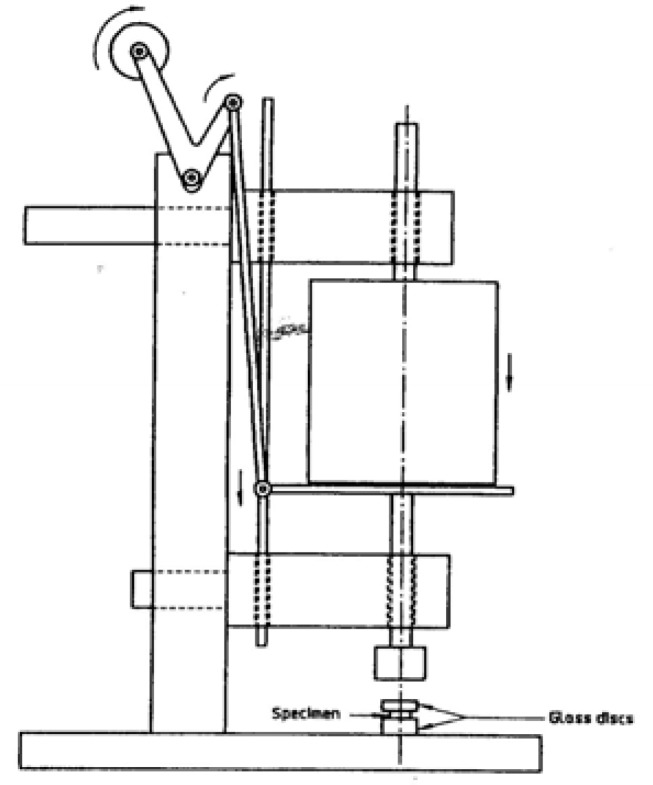
A schematic representation of loading device for measuring film thickness

The upper plate was removed and 0.1 ml of the mixed cement was placed in the center of the lower plate. This plate was placed centrally below the loading device (Figure 1). 

The second glass plate was replaced centrally on the cement, in the same orientation as in the original measurement. A vertical load of 150 N was applied on the top plate, 10 seconds before the end of the manufacturer’s stated working time. The load was applied smoothly and in such a manner that a minimum rotation occurs. At least 10 minutes after the application of the load, the plates were removed from the loading device and the thickness of the two glass plates, with the interposing cement, were measured once again with the digital micrometer. The film thickness for each specimen was measured as the difference in thickness of the two glass plates with and without cement. Each group had a sample size of five, from which the mean thicknesses were calculated. Film thickness was measured at 37C, 25C, and 10C for each material. 

**To measure flow rate**

To measure the relative flow rate of the cement in this study, a method described in ISO [12] and ADA [13] was used. Total of 0.5 mL of luting cement was placed on the center of a glass plate and a second glass plate of equal size was placed over the first, with an added weight centered on top for 1 minute. Therefore, the total weight acting on the specimen including the weight of glass plate was 120 grams. The added weight was then removed; the cement between the glass plates was light-cured through the top glass plate according to the manufacturer’s instruction. The diameter of each sample was measured using a digital micrometer, the average mean and standard deviations were then calculated. The test was repeated five times for each material. The flow rate was measured at 37C, 25C, and 10C. 

To evaluate the interaction between the material and the temperature in each of the two tests, a two-way ANOVA was carried out. To determine the inter-material differences for each test, the data were analysed using one-way ANOVA and Tukey’s test at a significance level of 0.05. A Pearson Correlation test was also conducted to determine if a relationship could be observed between the film thickness and the flow properties of all materials.

## Results

**Figure 2:**
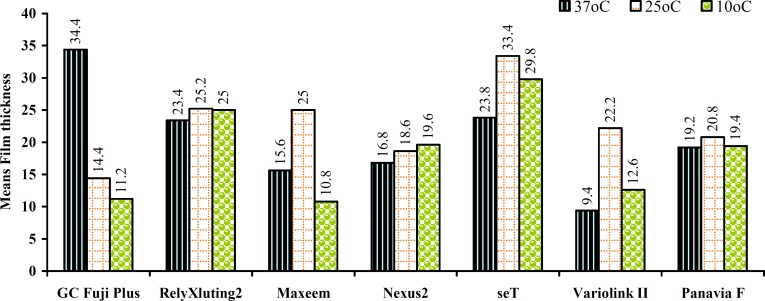
Means film thickness of all materials at 3 different temperatures

**Figure 3:**
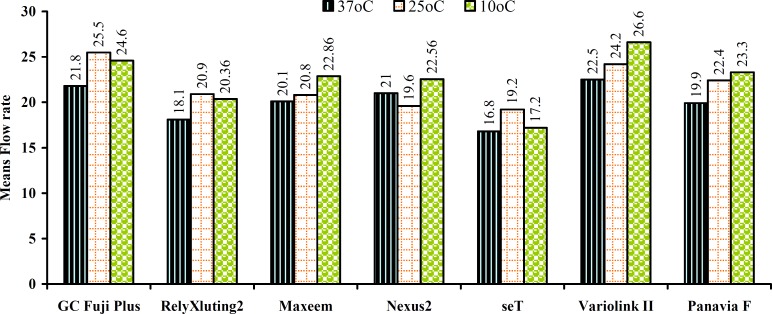
Means flow rate of all materials at 3 different temperatures

Both tested properties varied widely among materials (Table 2). The Pearson Correlation test showed a linear correlation between the film thickness and the flow rate for most of the materials that were supported by the temperature dependence of film thickness. Cooling the materials increased the fluidity of almost all the materials while the effect of temperature on the film thickness was material dependent. For instance, GC Fuji plus showed a sharp decrease in film thickness as the temperature decreased while Panavia F revealed very slight changes. Some others such as Nexus and RelyX showed a significant increase as the temperature decreased; seT and Maxcem exhibited a sharp increase at 25°C and a significant decrease at 10°C (Figures 2 and 3).

The mean film thickness at every temperature for all cements did not exceed 35µm. At 37°C the mean film thickness ranged from 9.4 (Variolink) to 23.8 (seT) except for the GC Fuji Plus which was 34.4. At 25°C, the mean film thickness ranged from 14.4 for GC Fuji Plus and 33.4 for seT; and at 10°C ranged from 10.8 for Maxcem to 29.8 for seT. However, there was a significant difference for some cements when comparing their film thickness at three different temperatures. With regard to the flow rate; the mean values at all temperatures within the cements and between the cements showed less diversity (Fig 4,5) than those of the film thickness. With regard to the temperature; the flow rate was varied from 16.79 (seT at 37°C) to 26.27 (Variolink at 20°C).

## Discussion

Based on the results of this study, the two hypotheses were rejected. The mean film thickness and its standard deviation is shown in Table 1. The effect of temperature on the film thickness was material dependent. Uneven values for film thickness within some cements resulted at different temperatures along with the large standard deviations. These results have been suggested to be related to the inadequate methods of testing for the film thickness, which resulted in the sliding and warping of the glass plates under a force of 150 N [3]. However, apart from the GC Fuji Plus at 37ºC and seT at 25ºC and 10ºC, all others showed a film thickness of less than 25µm which meets the requirement of ADA for resin cements. The results of this study indicate an implausible improvement in the film thickness of the new resin-based cements.

In the early days of resin-based cements, results of some studies showed that resin cements exhibited higher film thickness compared with the zinc phosphate cements [3.^4^]. White et al. [4] found in their study that some resin-based cements did not meet ADA specifications which have a maximal film thickness of 25µm whereas these samples exceeded beyond 40 µm. The authors speculated that the high viscosity influenced the film thickness. High viscosity resin-based materials set rapidly before they can flow sufficiently to achieve their minimum film thickness [4]. 

Along with recent advances in resin cements, in addition to the great adhesion to tooth structure, resin- based cements have superior mechanical properties and demonstrate increased retentive capabilities when compared to traditional cements [14-16]. They are used in conjunction with dentine bonding agents and can increase the fracture resistance of overlying ceramic materials [17]. It is reported that self-adhesive resin cements have similar or higher strength than conventional resin cements and also greater strength than resin-modified glass-ionomer cements [18-19].

**Table 2:** Mean values and standard deviations for film thickness and flow rate of resin luting cements in all temperatures

**Film thickness (µm)**				**Flow(mm)**		
**Temperature C°**				**Temperature C°**		
**Material**	**37**	**25**	**10**	**37**	**25**	**10**
GC Fuji Plus	^Aa^34.4±(3.9)	^Ab^ 14.4± (1.3)	^Ab^11.2± (3.6)	^Aa^21.8± (2.4)	^Ab^ 25.5± (2.4)	^Ab^ 24.6 ± (0.6)
RelyXluting2	^Ba^23.4± (2.2)	^B^ ^a^25.2± (2.5)	^Ba^25.0± (4.3)	^Ba^ 18.1± (0.7)	^Ba^ 20.9± (1.2)	^Ba^ 20.36 ± (1.1)
Maxeem	^Ca^15.6± (1.8)	^Bb^ 25.0± (3.1)	^Ac^ 10.8± (5.3)	^Aa^ 20.1± (1.6)	^Ba ^20.8± (1.6)	^Aa^ 22.86 ± (0.4)
Nexus2	^Ca^16.8± (4.4)	^Ca^18.6± (4.6)	^Ca^ 19.6± (1.1)	^Aa^ 21.0± (1.0)	^Ba^ 19.6± (0.7)	^Aa^ 22.56 ± (1.3)
seT	^Ba^23.8± (1.4)	^Db^ 33.4± (5.1)	^Bb^ 29.8± (3.3)	^Ba^ 16.8± (0.2)	^Ba^ 19.2± (0.7)	^Ba^ 17.2± (1.1)
Variolink ΙΙ	^Da^9.4± (2.4)	^BCb^ 22.2± (3.0)	^Aa^ 12.6± (2.5)	^Aa^ 22.5± (2.2)	^Aab^ 24.2± (3.1)	^Ab^ 26.6± (0.9)
Panavia F	^CBa^19.2± (2.2)	^BCa^ 20.8± (1.8)	^Ca^ 19.4± (2.2)	^Aa^ 19.9± (2.8)	^Ab^ 22.4± (1.0)	^Ab^ 23.3± (0.9)

Means with the same lower-case letter in each row were not significantly different (*p*> 0.05). Means with the same upper-case letter in each column were not significantly different (*p*> 0.05

On the other hand, when considering the film thickness, a recent study of new luting cements conducted by Kious et al. [9], found that the resin cements showed the thinner 25-μm standard of water-based cements with relative ease, therefore it does not seem necessary for clinicians to provide additional cement space. 

WU et al. [5] investigated the amount of cement space necessary for optimal seating of crowns cemented with the resin-based cements in comparison to the zinc phosphate cements. They found that seating discrepancy is inversely proportional to the cement space. The zinc phosphate cement required at least 40µm of cement space and the resin luting cements required 30 µm. Inadequate cement space resulted in crown seating disc-repancies of up to 364 µm which was not desirable [5]. 

Different temperatures were employed to assess if the resin-cements which were used directly from the fridge, very cold or hot storages, gave different results from the cements which were stored in the room temperature. This may be different compared with the powder-liquid materials which do not contain resin. Temperature of 10ºC induced the lowest film thickness for Maxcem and GC Fuji Plus than the other cements and this indicates that employing a low temperature for these materials would be advantageous when low film thickness is required. Conversely, a significant increase in the film thickness at 37°C was apparent for only GC Fuji Plus indicating that 37°C was not the optimal temperature for the lowest film thickness of this material.

Different parameters such as particle size, viscosity, fillers, and setting reactions may affect the film thickness of various classes of the adhesive resin cements. Among the resin-based cements in this study, regardless of temperature, the lowest film thickness was exhibited by Variolink with filler weight of 73.4%, followed by Maxcem (67%W), Nexus 2 (70%W), Panavia F (78%W) and seT (67%W). This order does not show a regular relationship between the filler content and the film thickness of the resin-based cements.

Van MeerBeek et al. in their study of clinically related properties revealed that no correlation was found between the maximum filler size and the film thickness or between the filler weight content and the consistency [3]. They also observed a high correlation between the consistency and the film thickness and concluded that consistency is the key factor which influences the film thicknesses of a dual cure luting composite. These authors speculated that the high consistency of the luting composites might adversely affect the optimal seating of the inlay [3]. 

The results of our study revealed an increase in the fluidity with decreasing temperature for almost all materials. An optimal flow rate of the luting agent determines the ease of manipulation and placement for the clinicians. White et al. [4] found that higher filler content elevates the viscosity and diminishes the flow. The rate of setting was also dependent on resin polymer size and smaller polymers generally set faster. However, according to the results of our study, we could not find any correlation between the filler weight and the flow rate of resin cements. For instance, Panavia F with the highest filler W% (78) showed a similar flow rate (21.88) to Maxcem (21.25) with the lowest filler W% (67). On the other hand, seT with the similar filler W% to Maxcem showed a significantly lower flow rate than Maxcem. 

An interesting phenomenon was observed when looking at the total values of the flow rate and the film thickness of the material. For instance, seT showed the lowest flow rate yet the highest film thickness. Similarly, Variolink displayed the highest flow value but exhibited the lowest film thickness. These results indicate an inverse relationship between the flow rate and the film thickness. These results do not agree with the results of Van Meerbeek [3] regarding the early-developed resin cements, where a linear correlation between their film thickness and consistency was reported.

The decrease in the viscosity, as the temperature decreased, can probably be attributed to the complexity of the stress distribution in a specimen during loading by the apparatus. The observed variation may also show a complex intrinsic change, occurring within the materials as they continue to mature. Further study with better suited apparatus is necessary to clarify and recognize what may occur in some of these materials. It must be emphasized that the results of the present study are valid for the presented laboratory conditions. Laboratory data may provide an insight into the clinical performance, however, a direct relationship between laboratory and clinical performance cannot always be assumed.

## Conclusion

Within the limitations of this study, the following conclusions were drawn. Differing in the temperature altered the film thickness and the flow properties of all materials to varying degrees. Cooling increased the fluidity of almost all materials while the effect of the temperature on the film thickness was material dependent. All tested luting cements exhibited a film thickness of less than 25µm at 37°C except for one RM-GIC (GC Fuji Plus=34.4). All but one self-adhesive resin cement (seT = 29 µm) had an average film thickness lower than 25µm. Comparable film thickness and flow for conventional and self-adhesive resin cements along with RM-GIC was observed. This in vitro study presents comparative clinical properties of seven resin-based cements, to help characterize cement types based on the effect of temperature and to ultimately aid in the clinical selection of dental cements.
